# Harlequin Syndrome after Thoracoscopic Repair of a Child with Tracheoesophageal Fistula (TEF)

**DOI:** 10.1055/s-0039-1697667

**Published:** 2019-09-26

**Authors:** Richard Wagner, Martin Lacher, Andreas Merkenschlager, Moritz Markel

**Affiliations:** 1 Klinik und Poliklinik für Kinderchirurgie, Universitätsklinikum Leipzig, Leipzig, Sachsen, Germany; 2 Klinik für Neuropädiatrie, Universitätsklinikum Leipzig Klinik und Poliklinik für Kinder- und Jugendmedizin, Leipzig, Sachsen, Germany

**Keywords:** Harlequin syndrome, esophageal atresia, thoracoscopic repair, neurocristopathy

## Abstract

Harlequin syndrome (HS) is a rare dysautonomia of the sympathetic nervous system leading to asymmetric facial flushing and sweating. In the literature, only a few cases of HS after thoracoscopic tracheoesophageal fistula (TEF) repair are reported. We report on a newborn with TEF who developed HS after thoracoscopic repair. On the first day of life, the girl (3,480 g, gestation age: 41 week) underwent thoracoscopic repair of a type C esophageal atresia (TEF; OR time 105 minute) without complications. The postoperative course was uneventful, the patient swallowed and thrived well and did not require esophageal dilatations. At 2 years of age, missing facial flushing, transpiration, and warming on the right side of her face during agitation were noticed. As no further intervention was required, the girl and her parents adapted well to the symptoms. Our report shows that the late onset of HS after the surgical procedure is unlikely a direct causal relation to the thoracoscopic operation but rather a shared embryological pathogenesis, like a neurocristopathy.

## Introduction

Harlequin syndrome (HS) is a very rare and seemingly benign condition characterized by unilateral loss of facial sympathetic functions. It was first described by Lance, an Australian professor of neurology, in 1988 in adults who showed unilateral flushing and sweating. He hypothesized that these syndromes occurred due to an idiopathic ipsilateral affection of the sympathetic outflow of the third root. 1

Since then, several reports and reviews have been published on this disorder. The cause of HS can be idiopathic and iatrogenic in both adults and children. 1 2 3 4 Most of the pediatric cases are related to a certain cause, for example, surgical procedure close to the autonomous nervous system in the upper chest, whereas the pathogenesis of congenital cases of the syndrome is still unclear. Leading to disturbance of autonomous functions like sweating and thermoregulation of the skin HS can be regarded as autonomous dysautonomia. It is different from other partial dysautonomias, although etiological and clinical overlap seems possible. 5 We report the third case of postoperative HS following thoracoscopic repair of a tracheoesophageal fistula (TEF). 6 7 8

## Case Report

**Fig. 1:**
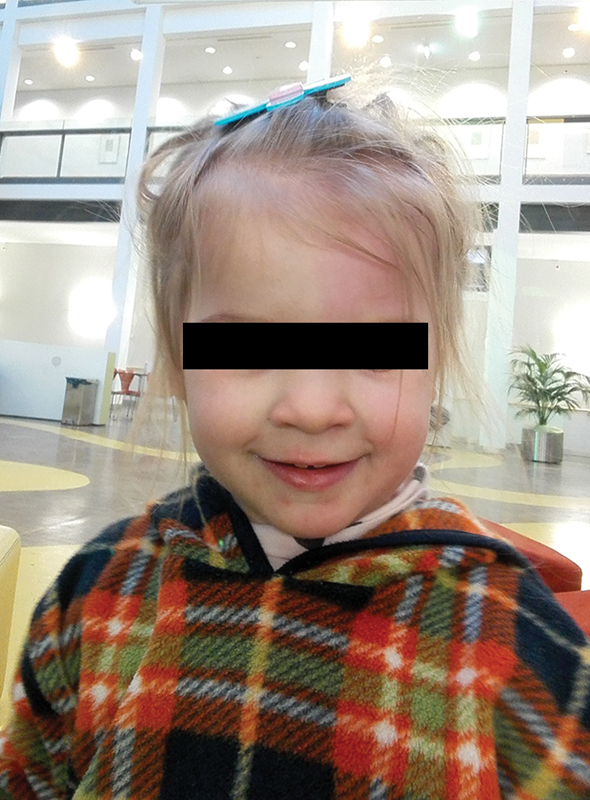
Girl with a loss of facial flushing and warming of the right side after exertion (age of 3 years; picture shown with parental approval).

A Caucasian baby girl of healthy parents was delivered spontaneously in the 41th week of gestation with a birth weight of 3,480 g, height of 50 cm, and head circumference of 37 cm. Postnatal she was diagnosed to have esophageal atresia (EA) type C (TEF). On the second day of life (DOL) and after bronchoscopy, the thoracoscopy was performed in left-sided half prone position. A type C (gross) EA with distal fistula (TEF) was confirmed. The azygos vein was electrically transected and the vagal nerve was preserved. After ligation of the fistula using a polyglactin 4.0 suture, a well-perfused, tension-less primary anastomosis was created with polyglactin 5.0 interrupted stiches and splinted with an 8 Charrière nasogastric feeding tube. A 12 Charrière chest-drain was placed. Operating time was 105 minutes. The girl returned to neonatal intensive care unit (NICU) and the postoperative course was uneventful. On the 2nd postoperative day (POD), nutrition was started via the feeding tube. On the 4th POD, the girl was extubated, and continuous positive airway pressure ventilation was performed until the 7th DOL. Oral feeds were started at the 7th DOL. At the 10th DOL, she was transferred to the pediatric surgical ward. The nasogastric feeding tube was removed at the 22nd DOL. The VACTERL workup revealed no associated malformations. A screening for metabolic diseases was unremarkable. The patient was discharged on the 25th DOL. The further development at home was uneventful. At the age of 1 year, the first endoscopic follow-up was performed and showed a wide esophageal lumen without stenosis. The girl had a normal height (P91) and weight (P81). The second endoscopic follow-up after 2 years was unremarkable too with a pH study showing slightly higher acid exposition without remarkable reflux episodes. At the age of 2 years, the girl presented with no complications, normal ingestion, normal weight, and normal height. However, her parents reported that the right side of her face remained pale and was not sweating during agitation ( Fig. 1 ). This was recognized for the first time at her second birthday. A detailed retrospective study of her NICU patient data and previous consultations revealed that this condition had not been observed before. The neurological and behavioral development of the patient was in accordance with her age. After 1 year of follow-up, the symptom of asymmetric sweating and paleness remains unchanged.

## Discussion

We report a case of HS that occurred 2 years after thoracoscopic repair of a child with TEF. HS is a repeatedly reported dysautonomia of the sympathetic innervation leading to loss of hemifacial flushing, sweating, and other sympathetic functions. It has to be separated from the Harlequin color change, which happens due to a paroxysmal change of vasomotor condition of the skin in the first DOLs, leading to alternating hemi-sided flushing. 9 In adults, the majority of cases cannot be related to any medical cause, while one-third of the patients appear due to expanding neoplasms or iatrogenic damage. 10 There are much fewer cases reported in children compared with adults. To date the majority of these patients have been described as having a secondary cause, although the limited number does not allow reliable conclusions. 3 11

To our knowledge, the report presented here describes the third patient of HS in a girl with EA (gross type C) and successful thoracoscopic treatment. This raises the question whether HS is a side effect of the thoracoscopic technique. Studies comparing the long-term complications after thoracoscopic versus open approaches for EA show no differences. 6 7 However, in 2009 Cozzi et al reported a case of hemifacial flushing and sweating after open repair of EA. And there has been a second report after open repair in 2018. 12 The other two cases of HS after repair of EA using thoracoscopy were reported in 2015. Interestingly, the first appearance of the hemifacial flushing happened between 1 and 3 years of age in all described patients. 8 12 13 Therefore, an intraoperative complication or lesion of the sympathetic nervous structures is unlikely due to the uneventful postoperative course and late onset of symptoms. The previously mentioned electrical division of the azygos vein is routinely performed by a lot of surgeons during the thoracoscopic approach to better expose the TEF. 14 As in the previously published case reports, the thoracoscopic repair of the EA did not include electric division of the azygos vein HS that cannot be explained by this operative step either. 8 Moreover, the relevant sympathetic nervous structures are localized further cranial from the surgical field during thoracoscopic EA repair. In reported cases of iatrogenic damage, the sympathetic structures were injured in the apex of the lung or even further cranial. 2 4 15

The pathogenesis of primary and secondary HS is still not sufficiently understood. The loss of hemifacial flushing and sweating most likely occurs due to a loss of function or dysregulation of the third-order neuron of the vaso- and sudomotor fibers of the ipsilateral sympathetic nervous pathway. 1 3 In our case and in previously reported ones, an obvious correlation between the surgical procedure and occurrence of symptoms cannot be found. This leads to the question whether there is a primary “pre-surgical” connection between HS and EA or the appearance in these cases is completely coincidental. 12

The theory of neurocristopathy describes the connection between disturbed migration of neural crest cells and maturational dysautonomia. Maldevelopment of the autonomic nervous system during embryogenesis can result in dysregulated sympathetic innervation. There is evidence for an association of EA and autonomic disturbances such as hyperhidrosis during feeding and hyperthermia. 12 16 17 Moreover, the association of EA with certain cardiovascular anomalies may suggest an underlying neurocristopathy of the caudal pharyngeal arch. 12 18 In this context, one case of HS associated with rare cardiovascular anomalies has been described. 19 It is tempting to speculate that both conditions, EA and HS, could be a result of disturbed neural crest migration. Due to the very limited number of reported cases of HS in children and in particular in association with EA, this conclusion is only hypothetical and further research addressing the pathogenesis of HS is needed.

## Conclusion

We report the third case of HS in a 3-year-old girl with EA after uncomplicated thoracoscopic repair. The late presentation of the HS in these cases makes a direct causal relation to the surgical procedure implausible. HS may therefore be a (primary) neurocristopathy associated with EA/TEF.

## Notes

New Insights and the Importance for the Pediatric Surgeon Harlequin syndrome is a rare occurrence after esophageal atresia repair with unknown etiology.
